# Price of Cocaine

**Published:** 1885-11-20

**Authors:** 


PRICE OF COCAINE.—We are glad to state that the price of Coca is reported greatly reduced from the enormous rate at first charged for it, having fallen from fifty cents a grain to about ten or fifteen cents.

